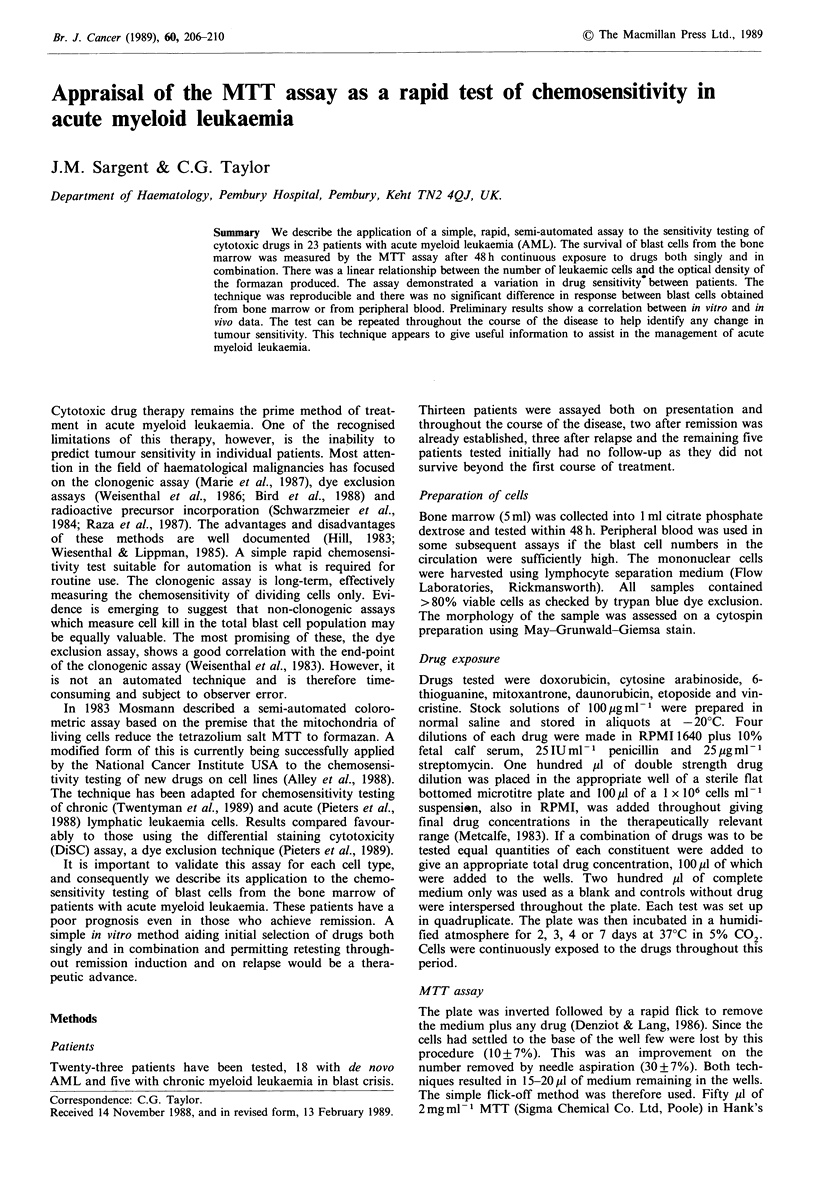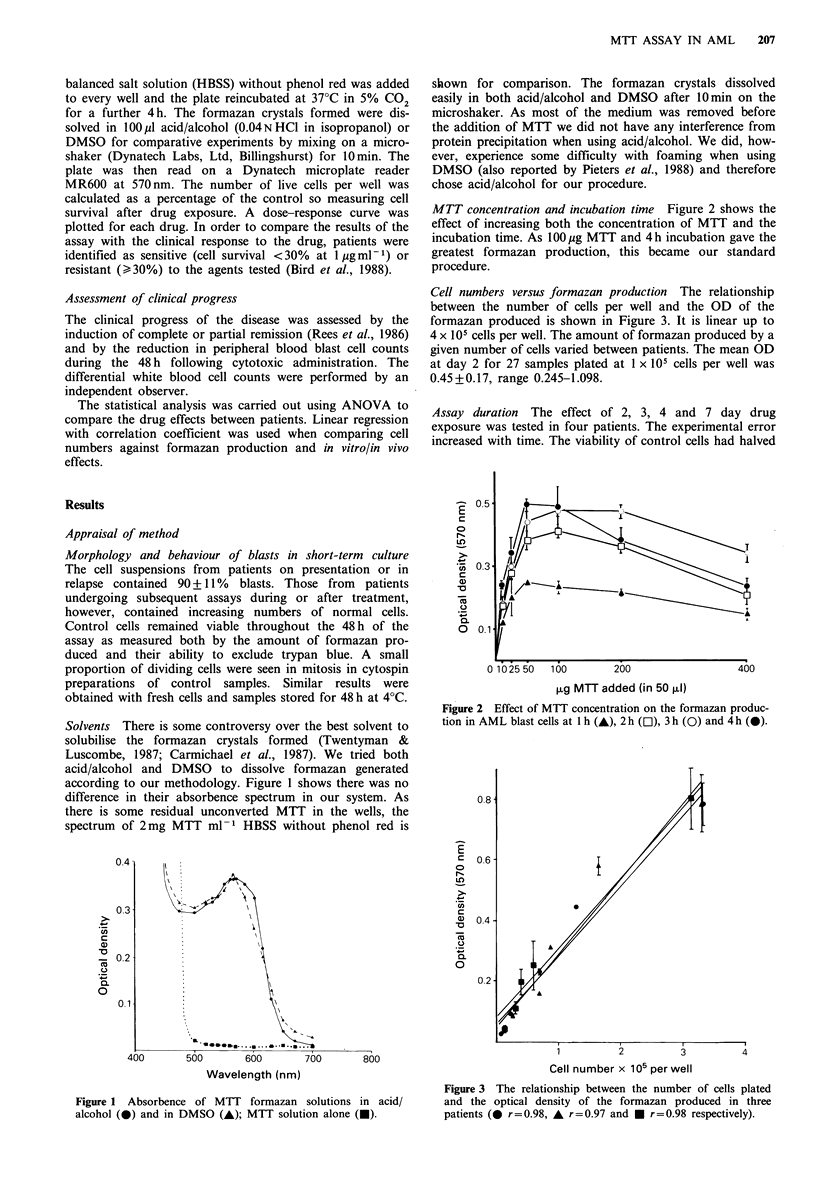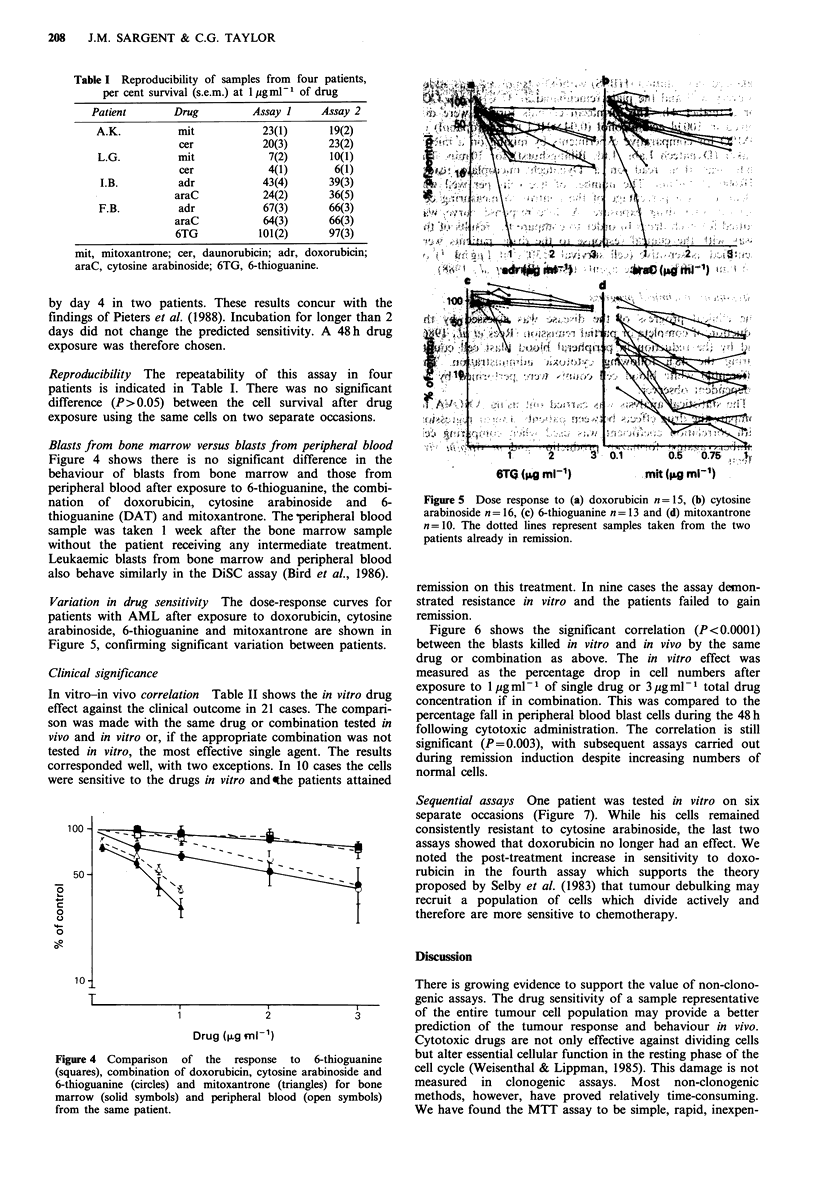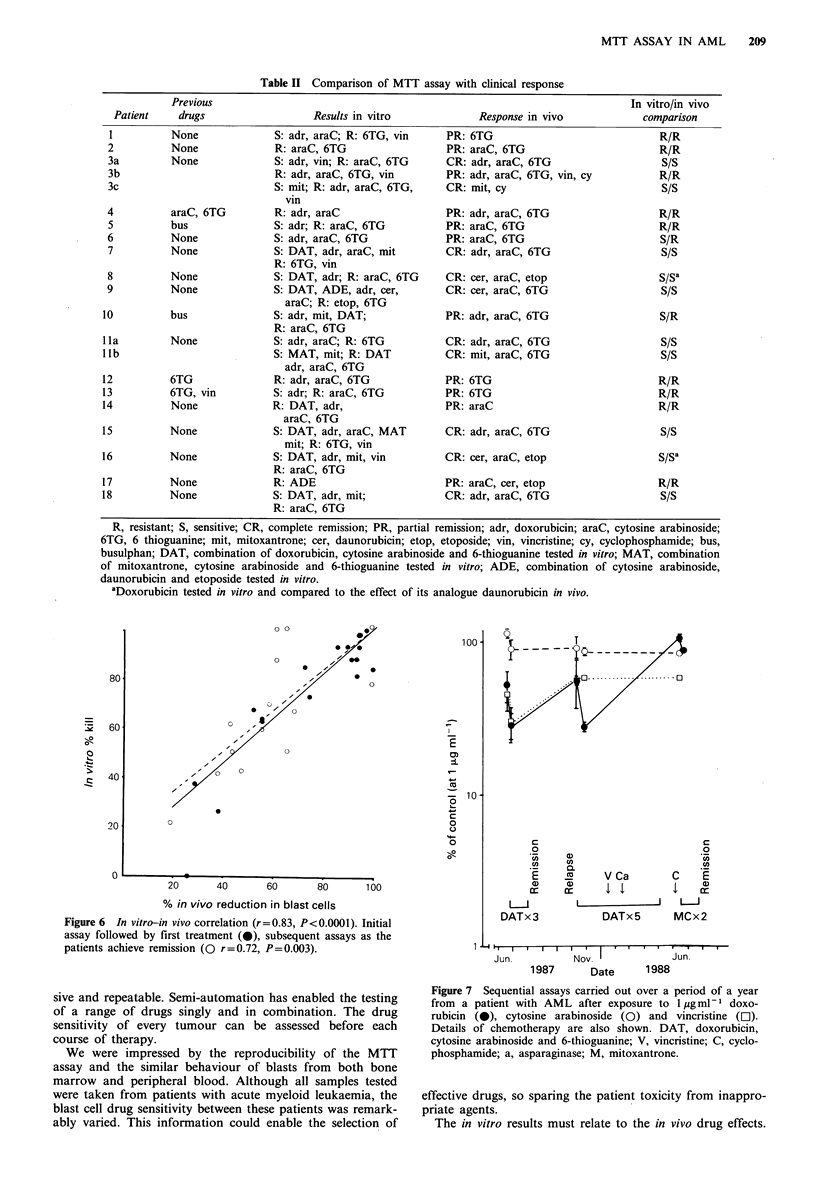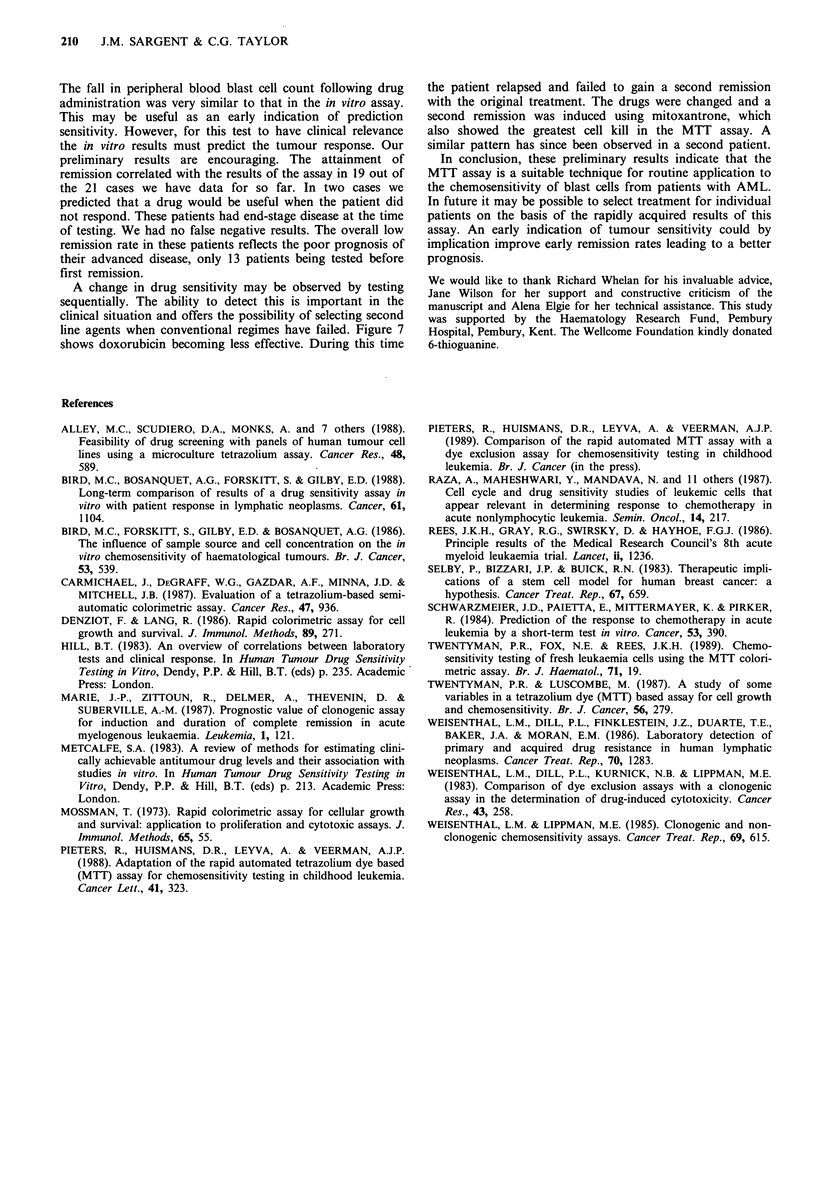# Appraisal of the MTT assay as a rapid test of chemosensitivity in acute myeloid leukaemia.

**DOI:** 10.1038/bjc.1989.252

**Published:** 1989-08

**Authors:** J. M. Sargent, C. G. Taylor

**Affiliations:** Department of Haematology, Pembury Hospital, Kent, UK.

## Abstract

We describe the application of a simple, rapid, semi-automated assay to the sensitivity testing of cytotoxic drugs in 23 patients with acute myeloid leukaemia (AML). The survival of blast cells from the bone marrow was measured by the MTT assay after 48 h continuous exposure to drugs both singly and in combination. There was a linear relationship between the number of leukaemic cells and the optical density of the formazan produced. The assay demonstrated a variation in drug sensitivity between patients. The technique was reproducible and there was no significant difference in response between blast cells obtained from bone marrow or from peripheral blood. Preliminary results show a correlation between in vitro and in vivo data. The test can be repeated throughout the course of the disease to help identify any change in tumour sensitivity. This technique appears to give useful information to assist in the management of acute myeloid leukaemia.


Br. J. Cancer (1989), 60, 206–210 © The Macmillan Press Ltd., 1989

### Appraisal of the MTT assay as a rapid test of chemosensitivity in acute myeloid leukaemia

J.M. Sargent & C.G. Taylor

*Department of Haematology, Pembury Hospital, Pembury, Kent TN2 4QJ, UK.*

**Summary** We describe the application of a simple, rapid, semi-automated assay to the sensitivity testing of cytotoxic drugs in 23 patients with acute myeloid leukaemia (AML). The survival of blast cells from the bone marrow was measured by the MTT assay after 48 h continuous exposure to drugs both singly and in combination. There was a linear relationship between the number of leukaemic cells and the optical density of the formazan produced. The assay demonstrated a variation in drug sensitivity between patients. The technique was reproducible and there was no significant difference in response between blast cells obtained from bone marrow or from peripheral blood. Preliminary results show a correlation between *in vitro* and *in vivo* data. The test can be repeated throughout the course of the disease to help identify any change in tumour sensitivity. This technique appears to give useful information to assist in the management of acute myeloid leukaemia.

Cytotoxic drug therapy remains the prime method of treatment in acute myeloid leukaemia. One of the recognised limitations of this therapy, however, is the inability to predict tumour sensitivity in individual patients. Most attention in the field of haematological malignancies has focused on the clonogenic assay (Marie *et al.*, 1987), dye exclusion assays (Weisenthal *et al.*, 1986; Bird *et al.*, 1988) and radioactive precursor incorporation (Schwarzmeier *et al.*, 1984; Raza *et al.*, 1987). The advantages and disadvantages of these methods are well documented (Hill, 1983; Wiesenthal & Lippman, 1985). A simple rapid chemosensitivity test suitable for automation is what is required for routine use. The clonogenic assay is long-term, effectively measuring the chemosensitivity of dividing cells only. Evidence is emerging to suggest that non-clonogenic assays which measure cell kill in the total blast cell population may be equally valuable. The most promising of these, the dye exclusion assay, shows a good correlation with the end-point of the clonogenic assay (Weisenthal *et al.*, 1983). However, it is not an automated technique and is therefore time-consuming and subject to observer error.

In 1983 Mosmann described a semi-automated colorometric assay based on the premise that the mitochondria of living cells reduce the tetrazolium salt MTT to formazan. A modified form of this is currently being successfully applied by the National Cancer Institute USA to the chemosensitivity testing of new drugs on cell lines (Alley *et al.*, 1988). The technique has been adapted for chemosensitivity testing of chronic (Twentyman *et al.*, 1989) and acute (Pieters *et al.*, 1988) lymphatic leukaemia cells. Results compared favourably to those using the differential staining cytotoxicity (DiSC) assay, a dye exclusion technique (Pieters *et al.*, 1989).

It is important to validate this assay for each cell type, and consequently we describe its application to the chemosensitivity testing of blast cells from the bone marrow of patients with acute myeloid leukaemia. These patients have a poor prognosis even in those who achieve remission. A simple *in vitro* method aiding initial selection of drugs both singly and in combination and permitting retesting throughout remission induction and on relapse would be a therapeutic advance.

### Methods

### *Patients*

Twenty-three patients have been tested, 18 with *de novo* AML and five with chronic myeloid leukaemia in blast crisis. Thirteen patients were assayed both on presentation and throughout the course of the disease, two after remission was already established, three after relapse and the remaining five patients tested initially had no follow-up as they did not survive beyond the first course of treatment.

Correspondence: C.G. Taylor.
Received 14 November 1988, and in revised form, 13 February 1989.

### *Preparation of cells*

Bone marrow (5 ml) was collected into 1 ml citrate phosphate dextrose and tested within 48 h. Peripheral blood was used in some subsequent assays if the blast cell numbers in the circulation were sufficiently high. The mononuclear cells were harvested using lymphocyte separation medium (Flow Laboratories, Rickmansworth). All samples contained >80% viable cells as checked by trypan blue dye exclusion. The morphology of the sample was assessed on a cytospin preparation using May–Grunwald–Giemsa stain.

### *Drug exposure*

Drugs tested were doxorubicin, cytosine arabinoside, 6-thioguanine, mitoxantrone, daunorubicin, etoposide and vincristine. Stock solutions of $100\,\mu g\,ml^{-1}$ were prepared in normal saline and stored in aliquots at −20°C. Four dilutions of each drug were made in RPMI 1640 plus 10% fetal calf serum, $25\,IU\,ml^{-1}$ penicillin and $25\,\mu g\,ml^{-1}$ streptomycin. One hundred $\mu l$ of double strength drug dilution was placed in the appropriate well of a sterile flat bottomed microtitre plate and $100\,\mu l$ of a $1\times10^6$ cells $ml^{-1}$ suspension, also in RPMI, was added throughout giving final drug concentrations in the therapeutically relevant range (Metcalfe, 1983). If a combination of drugs was to be tested equal quantities of each constituent were added to give an appropriate total drug concentration, $100\,\mu l$ of which were added to the wells. Two hundred $\mu l$ of complete medium only was used as a blank and controls without drug were interspersed throughout the plate. Each test was set up in quadruplicate. The plate was then incubated in a humidified atmosphere for 2, 3, 4 or 7 days at 37°C in 5% $CO_2$. Cells were continuously exposed to the drugs throughout this period.

### *MTT assay*

The plate was inverted followed by a rapid flick to remove the medium plus any drug (Denziot & Lang, 1986). Since the cells had settled to the base of the well few were lost by this procedure (10±7%). This was an improvement on the number removed by needle aspiration (30±7%). Both techniques resulted in 15–20 $\mu l$ of medium remaining in the wells. The simple flick-off method was therefore used. Fifty $\mu l$ of $2\,mg\,ml^{-1}$ MTT (Sigma Chemical Co. Ltd, Poole) in Hank's balanced salt solution (HBSS) without phenol red was added to every well and the plate reincubated at 37°C in 5% $CO_2$ for a further 4 h. The formazan crystals formed were dissolved in 100 μl acid/alcohol (0.04 N HCl in isopropanol) or DMSO for comparative experiments by mixing on a microshaker (Dynatech Labs, Ltd, Billingshurst) for 10 min. The plate was then read on a Dynatech microplate reader MR600 at 570 nm. The number of live cells per well was calculated as a percentage of the control so measuring cell survival after drug exposure. A dose–response curve was plotted for each drug. In order to compare the results of the assay with the clinical response to the drug, patients were identified as sensitive (cell survival <30% at 1 $\mu g\,ml^{-1}$) or resistant (⩾30%) to the agents tested (Bird *et al.*, 1988).

*Assessment of clinical progress*

The clinical progress of the disease was assessed by the induction of complete or partial remission (Rees *et al.*, 1986) and by the reduction in peripheral blood blast cell counts during the 48 h following cytotoxic administration. The differential white blood cell counts were performed by an independent observer.

The statistical analysis was carried out using ANOVA to compare the drug effects between patients. Linear regression with correlation coefficient was used when comparing cell numbers against formazan production and *in vitro/in vivo* effects.

### Results

*Appraisal of method*

*Morphology and behaviour of blasts in short-term culture* The cell suspensions from patients on presentation or in relapse contained 90±11% blasts. Those from patients undergoing subsequent assays during or after treatment, however, contained increasing numbers of normal cells. Control cells remained viable throughout the 48 h of the assay as measured both by the amount of formazan produced and their ability to exclude trypan blue. A small proportion of dividing cells were seen in mitosis in cytospin preparations of control samples. Similar results were obtained with fresh cells and samples stored for 48 h at 4°C.

*Solvents* There is some controversy over the best solvent to solubilise the formazan crystals formed (Twentyman & Luscombe, 1987; Carmichael *et al.*, 1987). We tried both acid/alcohol and DMSO to dissolve formazan generated according to our methodology. Figure 1 shows there was no difference in their absorbence spectrum in our system. As there is some residual unconverted MTT in the wells, the spectrum of 2 mg MTT $ml^{-1}$ HBSS without phenol red is shown for comparison. The formazan crystals dissolved easily in both acid/alcohol and DMSO after 10 min on the microshaker. As most of the medium was removed before the addition of MTT we did not have any interference from protein precipitation when using acid/alcohol. We did, however, experience some difficulty with foaming when using DMSO (also reported by Pieters *et al.*, 1988) and therefore chose acid/alcohol for our procedure.

**Figure 1** Absorbence of MTT formazan solutions in acid/alcohol (●) and in DMSO (▲); MTT solution alone (■).

*MTT concentration and incubation time* Figure 2 shows the effect of increasing both the concentration of MTT and the incubation time. As 100 μg MTT and 4 h incubation gave the greatest formazan production, this became our standard procedure.

*Cell numbers versus formazan production* The relationship between the number of cells per well and the OD of the formazan produced is shown in Figure 3. It is linear up to $4 \times 10^5$ cells per well. The amount of formazan produced by a given number of cells varied between patients. The mean OD at day 2 for 27 samples plated at $1 \times 10^5$ cells per well was 0.45±0.17, range 0.245–1.098.

*Assay duration* The effect of 2, 3, 4 and 7 day drug exposure was tested in four patients. The experimental error increased with time. The viability of control cells had halved

**Figure 2** Effect of MTT concentration on the formazan production in AML blast cells at 1 h (▲), 2 h (□), 3 h (○) and 4 h (●).

**Figure 3** The relationship between the number of cells plated and the optical density of the formazan produced in three patients (● $r=0.98$, ▲ $r=0.97$ and ■ $r=0.98$ respectively).

**Table I** Reproducibility of samples from four patients, per cent survival (s.e.m.) at 1 $\mu$g ml$^{-1}$ of drug

| *Patient* | *Drug* | *Assay 1* | *Assay 2* |
|---|---|---|---|
| A.K. | mit | 23(1) | 19(2) |
| | cer | 20(3) | 23(2) |
| L.G. | mit | 7(2) | 10(1) |
| | cer | 4(1) | 6(1) |
| I.B. | adr | 43(4) | 39(3) |
| | araC | 24(2) | 36(5) |
| F.B. | adr | 67(3) | 66(3) |
| | araC | 64(3) | 66(3) |
| | 6TG | 101(2) | 97(3) |

mit, mitoxantrone; cer, daunorubicin; adr, doxorubicin; araC, cytosine arabinoside; 6TG, 6-thioguanine.

by day 4 in two patients. These results concur with the findings of Pieters *et al.* (1988). Incubation for longer than 2 days did not change the predicted sensitivity. A 48 h drug exposure was therefore chosen.

*Reproducibility* The repeatability of this assay in four patients is indicated in Table I. There was no significant difference ($P>0.05$) between the cell survival after drug exposure using the same cells on two separate occasions.

*Blasts from bone marrow versus blasts from peripheral blood* Figure 4 shows there is no significant difference in the behaviour of blasts from bone marrow and those from peripheral blood after exposure to 6-thioguanine, the combination of doxorubicin, cytosine arabinoside and 6-thioguanine (DAT) and mitoxantrone. The peripheral blood sample was taken 1 week after the bone marrow sample without the patient receiving any intermediate treatment. Leukaemic blasts from bone marrow and peripheral blood also behave similarly in the DiSC assay (Bird *et al.*, 1986).

*Variation in drug sensitivity* The dose-response curves for patients with AML after exposure to doxorubicin, cytosine arabinoside, 6-thioguanine and mitoxantrone are shown in Figure 5, confirming significant variation between patients.

*Clinical significance*

In vitro–in vivo *correlation* Table II shows the *in vitro* drug effect against the clinical outcome in 21 cases. The comparison was made with the same drug or combination tested *in vivo* and *in vitro* or, if the appropriate combination was not tested *in vitro*, the most effective single agent. The results corresponded well, with two exceptions. In 10 cases the cells were sensitive to the drugs *in vitro* and the patients attained remission on this treatment. In nine cases the assay demonstrated resistance *in vitro* and the patients failed to gain remission.

**Figure 4** Comparison of the response to 6-thioguanine (squares), combination of doxorubicin, cytosine arabinoside and 6-thioguanine (circles) and mitoxantrone (triangles) for bone marrow (solid symbols) and peripheral blood (open symbols) from the same patient.

**Figure 5** Dose response to (**a**) doxorubicin $n=15$, (**b**) cytosine arabinoside $n=16$, (**c**) 6-thioguanine $n=13$ and (**d**) mitoxantrone $n=10$. The dotted lines represent samples taken from the two patients already in remission.

Figure 6 shows the significant correlation ($P<0.0001$) between the blasts killed *in vitro* and *in vivo* by the same drug or combination as above. The *in vitro* effect was measured as the percentage drop in cell numbers after exposure to 1 $\mu$g ml$^{-1}$ of single drug or 3 $\mu$g ml$^{-1}$ total drug concentration if in combination. This was compared to the percentage fall in peripheral blood blast cells during the 48 h following cytotoxic administration. The correlation is still significant ($P=0.003$), with subsequent assays carried out during remission induction despite increasing numbers of normal cells.

*Sequential assays* One patient was tested *in vitro* on six separate occasions (Figure 7). While his cells remained consistently resistant to cytosine arabinoside, the last two assays showed that doxorubicin no longer had an effect. We noted the post-treatment increase in sensitivity to doxorubicin in the fourth assay which supports the theory proposed by Selby *et al.* (1983) that tumour debulking may recruit a population of cells which divide actively and therefore are more sensitive to chemotherapy.

### Discussion

There is growing evidence to support the value of non-clonogenic assays. The drug sensitivity of a sample representative of the entire tumour cell population may provide a better prediction of the tumour response and behaviour *in vivo*. Cytotoxic drugs are not only effective against dividing cells but alter essential cellular function in the resting phase of the cell cycle (Weisenthal & Lippman, 1985). This damage is not measured in clonogenic assays. Most non-clonogenic methods, however, have proved relatively time-consuming. We have found the MTT assay to be simple, rapid, inexpen-

**Table II** Comparison of MTT assay with clinical response

| *Patient* | *Previous drugs* | *Results* in vitro | *Response* in vivo | In vitro/in vivo *comparison* |
|---|---|---|---|---|
| 1 | None | S: adr, araC; R: 6TG, vin | PR: 6TG | R/R |
| 2 | None | R: araC, 6TG | PR: araC, 6TG | R/R |
| 3a | None | S: adr, vin; R: araC, 6TG | CR: adr, araC, 6TG | S/S |
| 3b | | R: adr, araC, 6TG, vin | PR: adr, araC, 6TG, vin, cy | R/R |
| 3c | | S: mit; R: adr, araC, 6TG, vin | CR: mit, cy | S/S |
| 4 | araC, 6TG | R: adr, araC | PR: adr, araC, 6TG | R/R |
| 5 | bus | S: adr; R: araC, 6TG | PR: araC, 6TG | R/R |
| 6 | None | S: adr, araC, 6TG | PR: araC, 6TG | S/R |
| 7 | None | S: DAT, adr, araC, mit R: 6TG, vin | CR: adr, araC, 6TG | S/S |
| 8 | None | S: DAT, adr; R: araC, 6TG | CR: cer, araC, etop | S/S[a] |
| 9 | None | S: DAT, ADE, adr, cer, araC; R: etop, 6TG | CR: cer, araC, 6TG | S/S |
| 10 | bus | S: adr, mit, DAT; R: araC, 6TG | PR: adr, araC, 6TG | S/R |
| 11a | None | S: adr, araC; R: 6TG | CR: adr, araC, 6TG | S/S |
| 11b | | S: MAT, mit; R: DAT adr, araC, 6TG | CR: mit, araC, 6TG | S/S |
| 12 | 6TG | R: adr, araC, 6TG | PR: 6TG | R/R |
| 13 | 6TG, vin | S: adr; R: araC, 6TG | PR: 6TG | R/R |
| 14 | None | R: DAT, adr, araC, 6TG | PR: araC | R/R |
| 15 | None | S: DAT, adr, araC, MAT mit; R: 6TG, vin | CR: adr, araC, 6TG | S/S |
| 16 | None | S: DAT, adr, mit, vin R: araC, 6TG | CR: cer, araC, etop | S/S[a] |
| 17 | None | R: ADE | PR: araC, cer, etop | R/R |
| 18 | None | S: DAT, adr, mit; R: araC, 6TG | CR: adr, araC, 6TG | S/S |

R, resistant; S, sensitive; CR, complete remission; PR, partial remission; adr, doxorubicin; araC, cytosine arabinoside; 6TG, 6 thioguanine; mit, mitoxantrone; cer, daunorubicin; etop, etoposide; vin, vincristine; cy, cyclophosphamide; bus, busulphan; DAT, combination of doxorubicin, cytosine arabinoside and 6-thioguanine tested *in vitro*; MAT, combination of mitoxantrone, cytosine arabinoside and 6-thioguanine tested *in vitro*; ADE, combination of cytosine arabinoside, daunorubicin and etoposide tested *in vitro*.

[a]Doxorubicin tested *in vitro* and compared to the effect of its analogue daunorubicin *in vivo*.

**Figure 6** *In vitro–in vivo* correlation ($r=0.83$, $P<0.0001$). Initial assay followed by first treatment (●), subsequent assays as the patients achieve remission (○ $r=0.72$, $P=0.003$).

**Figure 7** Sequential assays carried out over a period of a year from a patient with AML after exposure to $1\,\mu g\,ml^{-1}$ doxorubicin (●), cytosine arabinoside (○) and vincristine (□). Details of chemotherapy are also shown. DAT, doxorubicin, cytosine arabinoside and 6-thioguanine; V, vincristine; C, cyclophosphamide; a, asparaginase; M, mitoxantrone.

sive and repeatable. Semi-automation has enabled the testing of a range of drugs singly and in combination. The drug sensitivity of every tumour can be assessed before each course of therapy.

We were impressed by the reproducibility of the MTT assay and the similar behaviour of blasts from both bone marrow and peripheral blood. Although all samples tested were taken from patients with acute myeloid leukaemia, the blast cell drug sensitivity between these patients was remarkably varied. This information could enable the selection of effective drugs, so sparing the patient toxicity from inappropriate agents.

The *in vitro* results must relate to the *in vivo* drug effects.

The fall in peripheral blood blast cell count following drug administration was very similar to that in the *in vitro* assay. This may be useful as an early indication of prediction sensitivity. However, for this test to have clinical relevance the *in vitro* results must predict the tumour response. Our preliminary results are encouraging. The attainment of remission correlated with the results of the assay in 19 out of the 21 cases we have data for so far. In two cases we predicted that a drug would be useful when the patient did not respond. These patients had end-stage disease at the time of testing. We had no false negative results. The overall low remission rate in these patients reflects the poor prognosis of their advanced disease, only 13 patients being tested before first remission.

A change in drug sensitivity may be observed by testing sequentially. The ability to detect this is important in the clinical situation and offers the possibility of selecting second line agents when conventional regimes have failed. Figure 7 shows doxorubicin becoming less effective. During this time the patient relapsed and failed to gain a second remission with the original treatment. The drugs were changed and a second remission was induced using mitoxantrone, which also showed the greatest cell kill in the MTT assay. A similar pattern has since been observed in a second patient.

In conclusion, these preliminary results indicate that the MTT assay is a suitable technique for routine application to the chemosensitivity of blast cells from patients with AML. In future it may be possible to select treatment for individual patients on the basis of the rapidly acquired results of this assay. An early indication of tumour sensitivity could by implication improve early remission rates leading to a better prognosis.

We would like to thank Richard Whelan for his invaluable advice, Jane Wilson for her support and constructive criticism of the manuscript and Alena Elgie for her technical assistance. This study was supported by the Haematology Research Fund, Pembury Hospital, Pembury, Kent. The Wellcome Foundation kindly donated 6-thioguanine.

### References

ALLEY, M.C., SCUDIERO, D.A., MONKS, A. and 7 others (1988). Feasibility of drug screening with panels of human tumour cell lines using a microculture tetrazolium assay. *Cancer Res.*, **48**, 589.

BIRD, M.C., BOSANQUET, A.G., FORSKITT, S. & GILBY, E.D. (1988). Long-term comparison of results of a drug sensitivity assay *in vitro* with patient response in lymphatic neoplasms. *Cancer*, **61**, 1104.

BIRD, M.C., FORSKITT, S., GILBY, E.D. & BOSANQUET, A.G. (1986). The influence of sample source and cell concentration on the *in vitro* chemosensitivity of haematological tumours. *Br. J. Cancer*, **53**, 539.

CARMICHAEL, J., DEGRAFF, W.G., GAZDAR, A.F., MINNA, J.D. & MITCHELL, J.B. (1987). Evaluation of a tetrazolium-based semi-automatic colorimetric assay. *Cancer Res.*, **47**, 936.

DENZIOT, F. & LANG, R. (1986). Rapid colorimetric assay for cell growth and survival. *J. Immunol. Methods*, **89**, 271.

HILL, B.T. (1983). An overview of correlations between laboratory tests and clinical response. In *Human Tumour Drug Sensitivity Testing in Vitro*, Dendy, P.P. & Hill, B.T. (eds) p. 235. Academic Press: London.

MARIE, J.-P., ZITTOUN, R., DELMER, A., THEVENIN, D. & SUBERVILLE, A.-M. (1987). Prognostic value of clonogenic assay for induction and duration of complete remission in acute myelogenous leukaemia. *Leukemia*, **1**, 121.

METCALFE, S.A. (1983). A review of methods for estimating clinically achievable antitumour drug levels and their association with studies *in vitro*. In *Human Tumour Drug Sensitivity Testing in Vitro*, Dendy, P.P. & Hill, B.T. (eds) p. 213. Academic Press: London.

MOSSMAN, T. (1973). Rapid colorimetric assay for cellular growth and survival: application to proliferation and cytotoxic assays. *J. Immunol. Methods*, **65**, 55.

PIETERS, R., HUISMANS, D.R., LEYVA, A. & VEERMAN, A.J.P. (1988). Adaptation of the rapid automated tetrazolium dye based (MTT) assay for chemosensitivity testing in childhood leukemia. *Cancer Lett.*, **41**, 323.

PIETERS, R., HUISMANS, D.R., LEYVA, A. & VEERMAN, A.J.P. (1989). Comparison of the rapid automated MTT assay with a dye exclusion assay for chemosensitivity testing in childhood leukemia. *Br. J. Cancer* (in the press).

RAZA, A., MAHESHWARI, Y., MANDAVA, N. and 11 others (1987). Cell cycle and drug sensitivity studies of leukemic cells that appear relevant in determining response to chemotherapy in acute nonlymphocytic leukemia. *Semin. Oncol.*, **14**, 217.

REES, J.K.H., GRAY, R.G., SWIRSKY, D. & HAYHOE, F.G.J. (1986). Principle results of the Medical Research Council's 8th acute myeloid leukaemia trial. *Lancet*, **ii**, 1236.

SELBY, P., BIZZARI, J.P. & BUICK, R.N. (1983). Therapeutic implications of a stem cell model for human breast cancer: a hypothesis. *Cancer Treat. Rep.*, **67**, 659.

SCHWARZMEIER, J.D., PAIETTA, E., MITTERMAYER, K. & PIRKER, R. (1984). Prediction of the response to chemotherapy in acute leukemia by a short-term test *in vitro*. *Cancer*, **53**, 390.

TWENTYMAN, P.R., FOX, N.E. & REES, J.K.H. (1989). Chemosensitivity testing of fresh leukaemia cells using the MTT colorimetric assay. *Br. J. Haematol.*, **71**, 19.

TWENTYMAN, P.R. & LUSCOMBE, M. (1987). A study of some variables in a tetrazolium dye (MTT) based assay for cell growth and chemosensitivity. *Br. J. Cancer*, **56**, 279.

WEISENTHAL, L.M., DILL, P.L., FINKLESTEIN, J.Z., DUARTE, T.E., BAKER, J.A. & MORAN, E.M. (1986). Laboratory detection of primary and acquired drug resistance in human lymphatic neoplasms. *Cancer Treat. Rep.*, **70**, 1283.

WEISENTHAL, L.M., DILL, P.L., KURNICK, N.B. & LIPPMAN, M.E. (1983). Comparison of dye exclusion assays with a clonogenic assay in the determination of drug-induced cytotoxicity. *Cancer Res.*, **43**, 258.

WEISENTHAL, L.M. & LIPPMAN, M.E. (1985). Clonogenic and nonclonogenic chemosensitivity assays. *Cancer Treat. Rep.*, **69**, 615.